# Mechanisms and research advances in mRNA antibody drug-mediated passive immunotherapy

**DOI:** 10.1186/s12967-023-04553-1

**Published:** 2023-10-04

**Authors:** Yuxiang Zhao, Linchuan Gan, Dangjin Ke, Qi Chen, Yajuan Fu

**Affiliations:** Fujian Key Laboratory of Innate Immune Biology, Biomedical Research Center of South China, College of Life Science, Fujian Normal University Qishan Campus, College Town, Fuzhou, Fujian PR China

**Keywords:** IVT mRNA, mRNA antibody, Passive immunotherapy, Nanomaterials

## Abstract

Antibody technology is widely used in the fields of biomedical and clinical therapies. Nonetheless, the complex in vitro expression of recombinant proteins, long production cycles, and harsh storage conditions have limited their applications in medicine, especially in clinical therapies. Recently, this dilemma has been overcome to a certain extent by the development of mRNA delivery systems, in which antibody-encoding mRNAs are enclosed in nanomaterials and delivered to the body. On entering the cytoplasm, the mRNAs immediately bind to ribosomes and undergo translation and post-translational modifications. This process produces monoclonal or bispecific antibodies that act directly on the patient. Additionally, it eliminates the cumbersome process of in vitro protein expression and extends the half-life of short-lived proteins, which significantly reduces the cost and duration of antibody production. This review focuses on the benefits and drawbacks of mRNA antibodies compared with the traditional in vitro expressed antibodies. In addition, it elucidates the progress of mRNA antibodies in the prevention of infectious diseases and oncology therapy.

## Introduction

mRNA is an unstable intermediate that bridges DNA and proteins, according to the central dogma theory, genetic information is first transmitted from DNA to mRNA and then from mRNA to proteins. In this step, mRNA works as a bridge to complete the process of conversion from DNA to proteins in an organism [1]. In 1992, a study reported that injecting insulin mRNA reversed diabetes insipidus in rats to some extent [2]. Initially, mRNA therapy was expected to replace or supplement the missing or defective proteins in patients. Later, mRNA was proposed to be used as an antigen in vaccines to treat cancer and other diseases, hence RNA vaccines were created [3–6]. Since the COVID-19 outbreak, enthusiasm for mRNA delivery proteins has increased to unprecedented levels. On December 11, 2020, the Food and Drug Administration (FDA) of the United States granted an emergency use license for the COVID-19 vaccine (BNT162b2) that was based on mRNA technology and developed by Biontech Co., Ltd. and Pfizer Pharmaceutical Co., Ltd. [7, 8]. Since then, mRNA vaccines have been widely used in human population and played an important role in preventing COVID-19 spread worldwide [9, 10]. Here we summarize some of the key events in the development of mRNA delivery technology (Table 1). With the use of mRNA delivery proteins, attempts are made to use in vitro transcribed (IVT) mRNAs to express antibodies to enhance the body’s resistance to epidemics, tumors, and toxins. Unlike mRNA vaccines that deliver antigens to trigger active immunity, mRNAs encoding antibodies directly elicit passive immunity [11–13]. In this review, we focus on the benefits and disadvantages of delivering antibodies *via* mRNAs and compared them with those of the traditional methods. Their perspectives are also discussed.

**Table 1:** Timeline of key advances in mRNA delivery technology

Time	Advances
1961	Discovery of mRNA [14].
1969	First in vitro translation of isolated mRNA [15].
1978	Delivery of mRNA by using liposomes [16].
1984	In vitro synthesis of biologically active RNA [17].
1989	Development of cationic lipid-mediated mRNA delivery [18].
1990	Demonstration that naked mRNA injected into mice is translated [19].
1992	Injection of mRNA into rats for the treatment of diabetes insipidus [2].
1993	Early reports of mRNA applications for infectious disease vaccines [20].
1995	Early reports of mRNA applications for cancer vaccines [21].
2001	First clinical trial of using mRNA-transfected dendritic cells in vitro [22].
2005	Demonstration that nucleotide modification can reduce the immunogenicity of RNAs [23].
2010	Generating iPSC by using mRNA [24].
2017	First testing of a personalized mRNA cancer vaccine in humans [25].
2020	COVID-19 mRNA Vaccines approved by FDA [8, 26].

## Advantages and challenges of IVT mRNA delivery systems

Transient transfection with plasmid as a vector and virus-mediated stable transfection are the main methods to introduce exogenous DNA fragments into cells to obtain new phenotypes. Nonetheless, with the advent of mRNA delivery, new methods are available for cells to express exogenous proteins [27–29]. Here, we list the potentials and limitations of mRNA delivery systems compared with the conventional methods.

### Advantages of IVT mRNA delivery systems

#### Advantages of IVT mRNA delivery systems compared with plasmid transfection

Plasmid transfection introduces a plasmid vector carrying exogenous DNA into a recipient cell, thereby inducing target gene overexpression in the cell. However, plasmid DNA is transcribed only during mitosis. Moreover, under normal circumstances, it is difficult for the plasmid vector to enter the nucleus. Hence, exogenous gene transcription is restricted, which significantly reduces the efficiency of protein translation. In contrast, since the mRNA delivery system is independent of the cell cycle, transient expression occurs by the cytoplasmic ribosomes; thus, it is a very efficient way to express proteins [27, 30, 31]. In addition, exogenous DNA introduction may activate the cGAS-STING signaling pathway in the cytoplasm and induce the production of type I interferons (IFN-I), thus triggering an immune response against the plasmid DNA. Besides, STING could inhibit the translation machinery to restrict the replication of diverse RNA viruses without expressing IFN-stimulated genes [32–35]. Whether or how STING influences the expression and translation of mRNA during IVT mRNA delivery is unclear and rneeds further investigation.

#### Advantages of IVT mRNA delivery systems compared with virus-mediated delivery

The genomes of certain viruses, such as lentiviruses, are modified to integrate exogenous genes into the host chromosome, thus causing stable protein expression. Infection with packaged viruses causes persistent transgene expression. Lentiviral vectors generally consist of two components, the packaging component and the vector component. In current systems, the original components of the virus are usually split onto different plasmids to ensure that the virus cannot be recombined. By co-transfecting packaging cells with multiple plasmids of the packaging and vector components, replication-defective lentiviral vector particles carrying the target gene can be harvested in the cell supernatant [29, 36–39]. However, since viruses randomly integrate into the host chromosomes, they increase the risk of genetic mutations. Additionally, when integrated into the tumor suppressor genes like P53, they increase the risk of cellular carcinogenesis in the host [40]. Contrastingly, since mRNA-mediated delivery is a non-integrated approach, RNA entering the nucleus is not required; thus, avoiding insertional mutagenesis caused by viral-mediated transfection.

Interestingly, certain viruses, such as the Sendai virus, use the non-integrating approach to transfer exogenous genes. Since their life cycle occurs entirely in the cytoplasm, they do not integrate into the host genome and are not affected by the cell cycle. However, residual viruses may hardly eradicate from cells [41]. While mRNAs have a short half-life in the cytoplasm and disappear gradually with cellular metabolism; hence, there is no need to specifically remove residual mRNAs [31, 42].

#### Advantages of IVT mRNA delivery systems compared with other protein delivery methods

Although recombinant proteins can be purified in vitro and directly imported into cells, their in vitro expression and purification is extremely tedious and complicated. Moreover, being a biological macromolecule, proteins are difficult to penetrate the cell membrane, and hence require physicochemical methods, such as electroporation or liposome encapsulation, to enter cells. Further, they may not reach their cytosolic targets even if they successfully cross the cell membrane. This may be because a significant portion of the proteins get trapped in vesicular structures, such as endosomes, and hence are unable to exert biological activity [28, 43–45].

### Challenges of IVT mRNA delivery systems

**Fig. 1:**
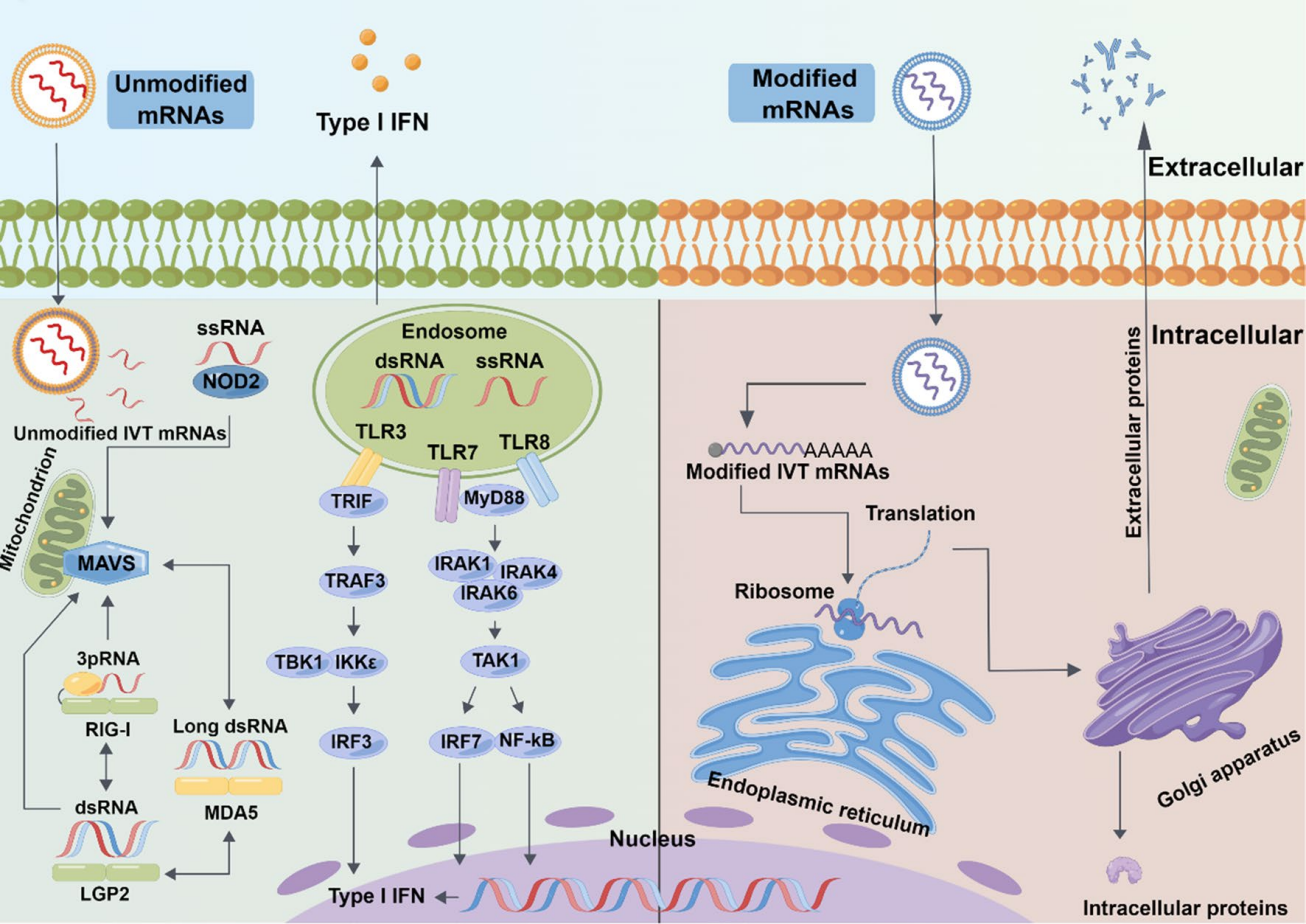
Unmodified IVT mRNAs can trigger an immune response in the body. Unmodified mRNAs have high immunogenicity that induces interferon production through activating TLR3, TLR7, and TLR8. However, in vitro modifications of mRNAs can reduce their immunogenicity to avoid the risk of triggering an immune storm in the organism

Despite the advantages, several shortcomings limit the development and application of the mRNA delivery system. (1) mRNAs are easily degraded by nucleases in the cell or environment since they are single-stranded and less stable than double-stranded DNA. When mRNAs enter the cytoplasm, they get hydrolyzed; hence, the mRNA-delivered system cannot express long-lived proteins. (2) Since mRNAs are negatively charged, they do not easily pass through the negatively charged cell membrane. Therefore, they require specific carriers to cross the cell membrane. (3) Unmodified mRNAs may stimulate RNA receptors including RIG-I like receptors (RIG-1, LGP-2 or MDA-5) and toll-like receptors (TLR3, TLR7, or TLR8), thereby eliciting immunogenic responses and unavoidable deleterious side effects (Table 2) [23, 46–51]. Despite the existing challenges, obstacles of IVT mRNAs, such as mRNA degradation and high immunogenicity, have been gradually solved in recent years through in vitro modifications of mRNAs and optimization of mRNA purification methods. Simultaneously, the development of the nano-delivery technology enables mRNA encapsulation that promotes cellular uptake (Fig. 1) [52]. Henceforth, we focus on the status of IVT mRNA research and outline the modification, purification, and delivery methods for IVT mRNAs.

**Table 2:** Human TLRs and their major ligands

TLRs	Ligands
TLR1	Triacylated lipopeptides
TLR2	Peptidoglycans, lipoproteins, lipopeptides, phospholipoman, lipoarabinomannan, porins
TLR3	dsRNA
TLR4	Lipopolysaccharide, envelope proteins, heat-shockproteins
TLR5	Flagellin
TLR6	Zymosan, diacylated lipopeptides
TLR7	ssRNA
TLR8	ssRNA
TLR9	CpG-DNA, hemozoin
TLR10	Unclear

## Modification, purification, and delivery of IVT mRNA

### Modifications of IVT mRNAs

Post-transcriptional modifications of eukaryotic mRNAs are complex. A mature mRNA consists of a 5′ cap, a 5′ untranslated region (5′ UTR), a coding region, a 3′ untranslated region (3′ UTR), and a 3′ polyadenylate tail. The modifications shield mRNA from degradation by exonucleases. However, IVT mRNAs are transcribed from a segment of linear DNA and lack post-transcriptional modifications that directly affects mRNA stability and protein translation [53–55]. For example, 7-methylguanosine is attached to the first transcribed nucleotide through a 5′, 5′-triphosphate bond and protects mRNA from 5′-3′ exonucleases. Thus, the 5′ cap protects mRNA from RNase degradation. Moreover, the translation system in the cytoplasm recognizes the cap structure through the cap-binding complex (CBP). This helps the small ribosomal subunit bind to mRNA and recognize the start codon to initiate translation. Without the cap structure, CBP cannot bind to mRNA, thus translation efficiency is greatly reduced. [56–59] Therefore, artificially introducing the cap structure in IVT mRNAs can enhance their translation, improve their stability, reduce their immunogenicity, and extend their half-life. Similarly, adding the 3′ tail and modifying special bases of IVT mRNAs increase their stability and reduce their immunogenicity to avoid immune response caused by TLRs activation [60–64]. The sequences of COVID-19 mRNA vaccines are optimized to reduce the production of proinflammatory type I interferons [9]. For example, the uridine is replaced with purified N1-methyl-pseudouridine (1MΨ); mRNA vaccine BNT162Tech contains human α-globin RNA with optimized Kozak sequence in 5′ UTR domain, whereas mRNA vaccine CVnCoV contains artifacts from restriction and transcription site plus Kozak sequence [65].

### Purification methods of IVT mRNAs

Although capping or incorporating modified nucleotides can greatly reduce the immunogenicity of mRNAs, it is hard to achieve 100% modification efficiency in vitro [66]. The unmodified mRNAs may trigger cytokine storm in the body. In addition, double-stranded RNA (dsRNA) may occur as a transcriptional byproduct and provoke an innate immune response by RNA sensors. Additionally, the remaining process-related impurities, such as protease residues, DNA templates, organic solvents, and metal ions, need to be removed. Therefore, methods for large-scale production and purification of mRNAs are critical. Common RNA purification methods are polyacrylamide gel electrophoresis, ultracentrifugation, ion exchange chromatography, hybridization affinity chromatography, reversed-phase chromatography, and HPLC-based purification methods [67–72]. These processes ensure safety for subsequent in vivo experiments. Moreover, attempts are made to reduce the immunogenicity of mRNAs and the formation of by-products for avoiding the subsequent tedious purification methods. For instance, Wu et al. used thermostable T7 RNAPs to synthesize functional mRNAs that had reduced immunogenicity and did not require a post-synthesis purification step [73]. Similarly, Xia et al. used the psychrophilic phage VSW-3 RNA polymerase to reduce terminal and full-length dsRNA byproducts in vitro transcription [74]. In conclusion, mRNA drugs should be administered with minimum innate immune effects to avoid subsequent troubles.

### Delivery mode of IVT mRNAs

**Fig. 2:**
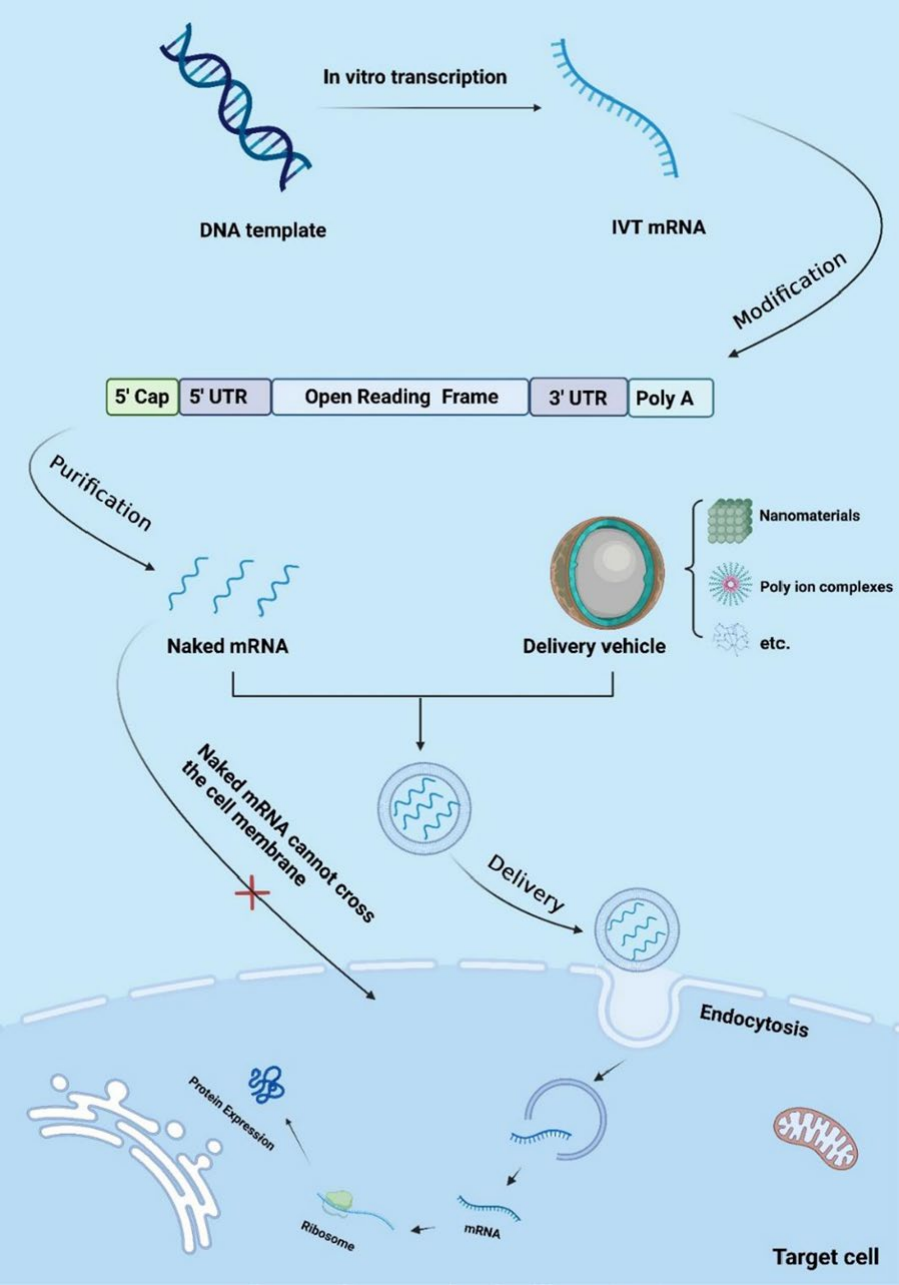
Modification, purification, and delivery of IVT mRNA. In vitro transcribed mRNAs are delivered to target cells via vehicle after modifications such as capping and are translated into proteins in the cytoplasm

mRNAs have a negative charge and large molecular weight, hence do not easily cross the cell membrane [75, 76]. Recently, encapsulation with lipid nanoparticles (LNPs) is undoubtedly the most popular delivery method [77, 78]. The BNT162b2 (Pfizer/BioNTech) and mRNA-1273 (Moderna) vaccines, approved by the FDA, are mRNA vaccines encapsulated with LNPs [30, 79]. Electroporation, protamine, cationic nanoemulsion, and cationic polymer liposomes are the common delivery methods [3, 80, 81]. Selecting the appropriate vectors can effectively avoid RNA degradation, improve RNA presentation efficiency and biosafety, and promote the clinical translation of mRNA therapies (Fig. 2) [75, 82–84]. Here, we have compiled a selection of methods used by researchers in mRNA delivery, which contains the composition, size and type of nanoparticles used (Table 3).

**Table 3:** Nanomedical approaches to mRNA delivery

Application	Main components of vector	Size (nm)	Route	mRNA dosage	Status	References
mRNA vaccine	ALC-0315	Not found	I.M.	30 µg (For humans)	Market	[8]
	SM-102	Not found	I.M.	50 µg (For humans)	Market	[26]
	Cholesterol, phosphatidylserine	< 200	S.C.	12 µg	Completed	[20]
Protein replacement	PEG-PAsp(DET)	100	I.C.V.	3 µg	Completed	[85]
	Biodegradable ionizable lipids (ATX)	Undisclosed	I.V.	2 and 4 mg/kg	Completed	[86]
	Phospholipids, cholesterol, PEG	< 100	I.V.	190 and 760 ng	Completed	[87]
mRNA antibody	DSPC, cholesterol and PEG-lipid	Undisclosed	I.V.	0.1, 0.3 and 0.6 mg/kg	Active(Phase I)	[88]
	DOTAP, cholesterol	183–339	I.T.	5 µg	Completed	[89]
	DSPC, cholesterol and PEG-lipid	88.99	I.V.	0.2 and 1 mg/kg	Completed	[90]
	TransIT-mRNA Transfection kit	Undisclosed	I.V.	5 µg	Completed	[91]
Gene delivery and editing	Cationic ionizable lipids, phospholipids, cholesterol	150	I.V.	0.1 mg/kg	Completed	[92]
	306-O12B (A leading tail-branched bioreducible lipidoid)	110	I.V.	3 mg/kg	Completed	[93]

*S.C.* subcutaneous injection, *I.C.V.* intracerebroventricular administration, *I.V.* intravenous injection, *I.T.* intratracheally administration

## Applications of mRNA delivery in passive immunization

**Fig. 3:**
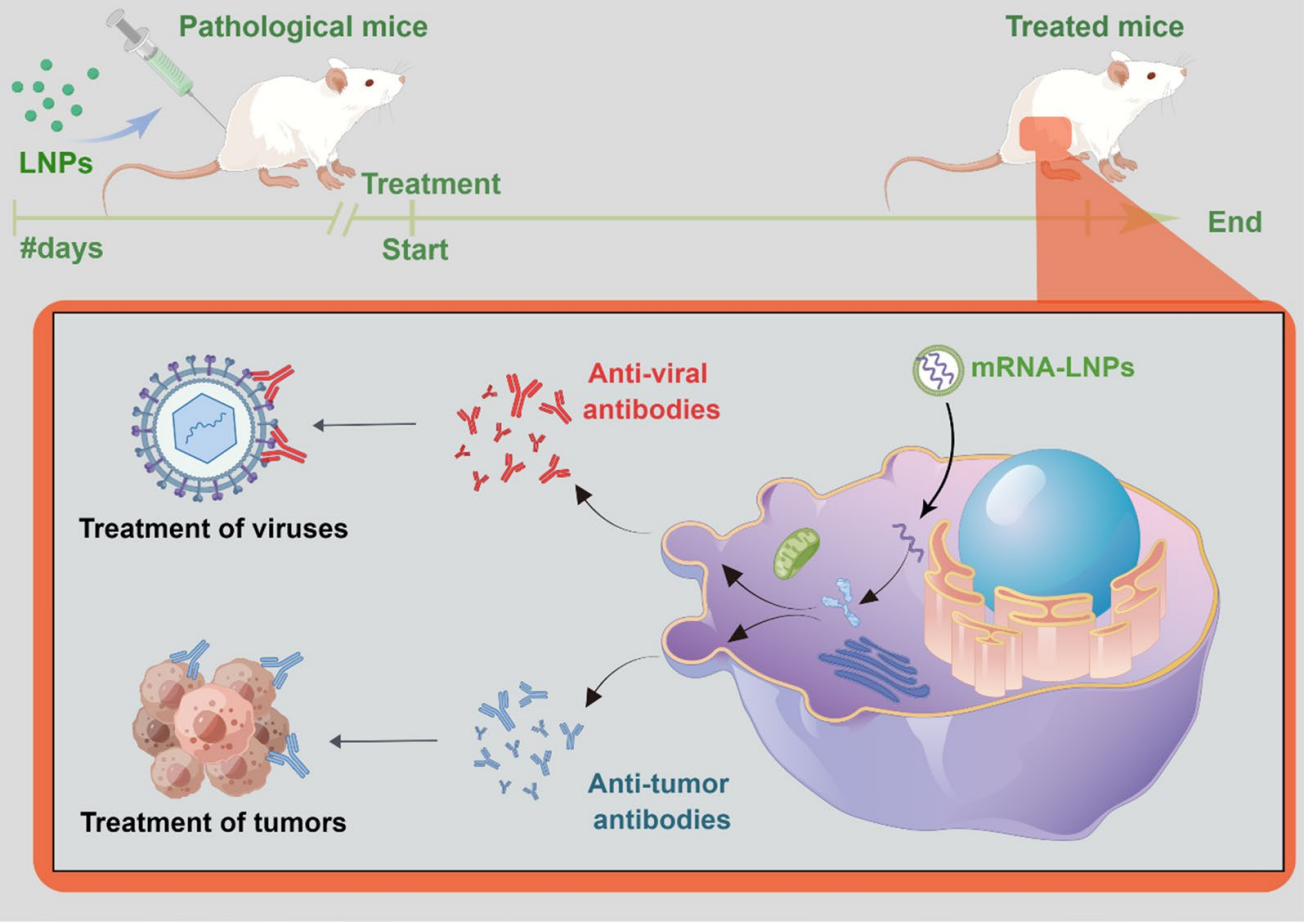
Schematic illustration of the mRNA antibody treatment. After being injected into the body, the mRNAs enter the cell with carriers such as LNP and bind to ribosomes to initiate translation. The antibodies produced are secreted by the cells in the extracellular compartment and travel via blood circulation to all body parts to exert their effects

Strong immunogenicity and instability have been the major constraints to the application of mRNA delivery for a long time which have largely been overcome by technological advances. Multiple mRNA vaccines against infectious diseases and cancers showed encouraging results in animal models. Several mRNA vaccines against COVID-19 played a pivotal role in curbing the spread of human epidemics. Briefly, mRNA vaccines induce adaptive immunity in humans by delivering mRNAs encoding antigens. Recently, researchers have attempted to directly deliver mRNAs encoding antibodies to enable the body to acquire adaptive immunity (Fig. 3; Table 4). In contrast to mRNA vaccines, mRNA-encoded antibodies exhibit rapid response. Once introduced into the body, they do not have an incubation period; hence, the protective effects are immediate. In this section, we describe the recent advances in mRNA-encoded antibodies.

### Defense against viruses by delivering mRNAs encoding antibodies

#### Defense against SARS-CoV-2 by mRNA-encoded antibodies

**Fig. 4:**
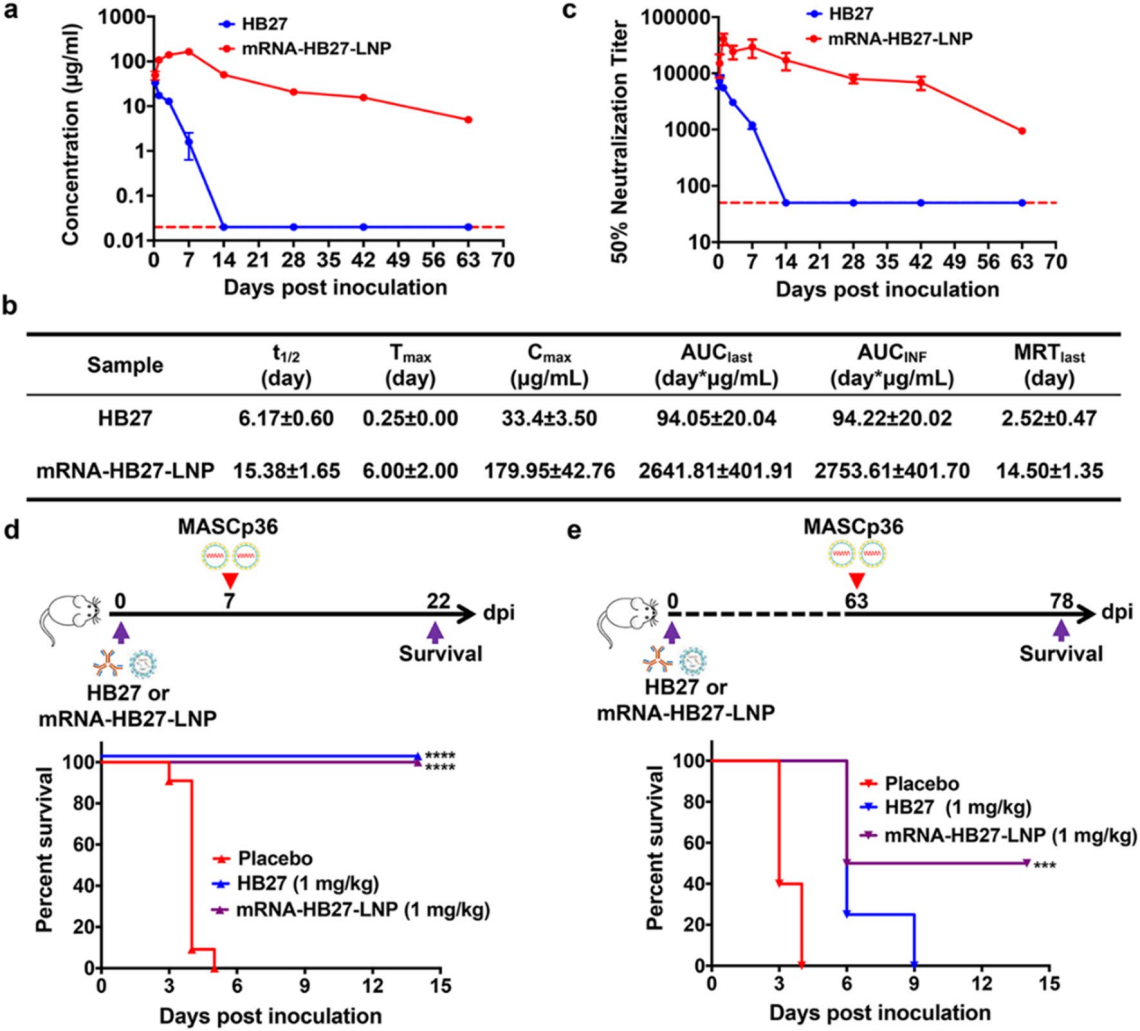
mRNA-HB27-LNP provides a long-term protection against SARS-CoV-2 challenge in mice. **a** The antibody concentration of serum in mice by ELISA. Briefly, groups of 6–8-week-old ICR mice were i.v. administrated with a single dose of 1 mg/kg of HB27 (n = 4) and HB27-mRNA-LNP (n = 4), respectively. At indicated times post administration, sera of mice were measured by ELISA. Dotted lines indicate the limits of detection. **b** Analysis of antibody pharmacokinetics in serum after the i.v. administration with a single dose of HB27 and mRNA-HB27-LNP. Calculations were performed using WinNolin. **c** NT50 of serum in mice by VSV-based SARS-CoV-2 pseudovirus. Data are shown as mean ± SEM. Dashed lines represents limit of detection. **d, e** Experimental design. Briefly, groups of 8-month-old female BALB/c mice were i.v. administrated with a single dose of 1 mg /kg of HB27 or mRNA-HB27-LNP (n = 4 or 5) and Placebo (n = 5). Then at 7 days or 63 days post administration, mice were challenged with 6 × 103 PFU of MASCp36, respectively, and the clinical symptoms and mortality were recorded for 14 days. Survival curves of mice after lethal challenge by MASCp36 at 7 days (**d**) and 63 days (**e**) after the i.v. administration. Data were analyzed by Wilcoxon log-rank survival test (**P < 0.01). **a-e** Reproduced with permission from ref [90]. Copyright © 2022, The Author(s)

Globally, > 630 million people were infected with the SARS-CoV-2 virus and the death toll reached 6.6 million by early December 2022. This epidemic swept the world and adversely affected human life [94–98]. Qin et al. encoded light and heavy chain mRNAs of HB27, a SARS-CoV-2 neutralizing antibody, encapsulated them in LNPs (mRNA-HB27-LNP), and successfully expressed biologically active antibodies in mice. Strikingly, mRNA-HB27-LNP has a longer circulating half-life and better prophylactic effect than the original HB27 protein. Moreover, in mice, intravenous administration of a single dose of mRNA-HB27-LNP was effective against lethal doses of MASCp36, a mouse-adapted SARS-CoV-2 strain, and did not cause significant adverse effects. After a single injection of 1 mg/kg mRNA-HB27-LNP, the antibody concentration in mice reached a maximum on day 7. The serum antibody concentration remained at 4.95 µg/mL with a mean of 179.95 µg/mL at 63 days after administration, a result that was much higher than that of an equal dose of original HB27 antibody in protein format (Fig. 4). Furthermore, mRNA-HB27-LNP is highly potent against the beta variant of SARS-CoV-2. Interestingly, prophylactic administration of mRNA-HB27-LNP protected animals in a close contact transmission model and provided long-term prophylactic efficacy against SARS-CoV-2 infection [90, 99]. Similarly, using the Venezuelan equine encephalitis virus (VEEV) replicon, Ye et al. constructed a single mRNA vector expressing both, the heavy and light chains, of the CB6 monoclonal antibody (VEEV-rep-CB6) by an alphavirus replicon particle (VRP) delivery system. The VEEV-VRP is an ideal delivery system as it has a broad range of susceptible host cells and high expression level of cytoplasmic proteins. Studies revealed that the local delivery of CB6 antibody-encoding mRNAs through intranasal administration induced antibody expression in multiple cell lines in mouse lungs, effectively blocked SARS-CoV-2 infection, reduced viral titers, and decreased damage to mouse lung tissues [100, 101].

#### Defense against HIV by mRNA-encoded antibodies

VRC01 is a neutralizing anti-HIV-1 antibody [102–105]. Weissman et al. evaluated the kinetics and protective effects of the 1-methyl pseudouridine (m1Ψ)-modified and FPLC-purified mRNA-based delivery of the VRC01 antibody. Mice administered 1.4 mg/kg mRNA resulted in VRC01 antibody concentrations in plasma of 170 mg/mL at 24 h. A single intravenous injection of mRNA-VRC01-LNPs maintained high antibody levels for 5 days in mice. While successive injections of mRNA-VRC01-LNPs maintained high antibody levels for a long time, with no obvious immune inflammatory response in mice triggered by the purified mRNA-VRC01-LNPs. To determine whether mRNA-VRC01-LNPs protect animals from the HIV-1 virus, mice primed with VRC01 mRNAs to achieve high antibody titers were injected with SF162 HIV-1. The results demonstrated that HIV-1 replication was robustly inhibited. Similar attack experiments by HIV-1 JR-CSF also supported that mRNA-encoded antibodies effectively protect the organism from viral invasion [106]. Recently, some camelid antibodies have been developed against HIV [107]. Nanobodies are found in the blood of camelids and sharks, which contain only a heavy chain variable region (VHH) and two conventional CH2 and CH3 regions. VHH has the properties of high solubility, low aggregation, and resistance to high temperatures, strong acids and bases, which has a promising prospect for the development of therapeutic antibody drugs [108]. However, there wasn’t any research yet on treating HIV with mRNA-encoded camelid antibodies, which might be a new therapeutic option.

#### Defense against influenza a virus by mRNA-encoded antibodies

mRNAs can be used to deliver specific monoclonal antibodies as well as bispecific antibodies. Compared to conventional monoclonal antibodies, bispecific antibodies can bind two different antigenic epitopes, providing broader therapeutic effects. They could enhance the ability of immune cells to kill target cells because they could target both cell types [109–112]. Saelens et al. developed a bispecific VHH antibody (RiboBiFE) that specifically binds to the activating mouse Fcg receptor IV (FcgRIV) and the ectodomain of the conserved influenza A matrix protein 2 (M2e). Thus, the antibody selectively recruits the innate immune cells to influenza A virus-infected cells [113, 114]. The bispecific VHH antibodies were successfully produced in mice lungs by formulating the mRNA encoding RiboBiFE into DOTAP/cholesterol nanoparticles and intrabronchial drops. The mRNA-RiboBiFE was present for a greater duration in the lungs compared with the protein-based RiboBiFE. Mice treated with mRNA-RiboBiFEs were significantly better protected against death caused by influenza virus infection compared to negative controls. In addition, these bispecific VHH antibodies greatly reduced the morbidity caused by influenza A virus attack, which provides a new strategy against them [89, 108, 115, 116].

#### Defense against Zika virus by mRNA-encoded antibodies

Zika virus (ZIKV) is a single-stranded RNA virus that primarily transmits via mosquitoes [117–119]. ZIKV-117 is a potent neutralizing mAb with broad activity against the African and Asian lineages of ZIKV [120]. Van Hoeven N et al. intramuscularly injected mRNA-encoded ZIKV-117 to combat ZIKV transmission. For effective expression of mRNA antibodies in vivo, researchers used a replicating viral RNA (ZIKV-117 repRNA) to amplify the antibody-encoding mRNAs. The repRNA increased the serum antibody concentration by > 30-fold in mice than non-replicating mRNA. In the lethal ZIKV challenge model in mice, the ZIKV-117 repRNA provided effective protection to mice. This study provides a possible scenario for future human deployment of inhibitory antibodies to curb the spread of epidemics [121].

#### Defense against chikungunya virus by mRNA-encoded antibodies

Chikungunya virus (CHIKV) causes an acute infectious disease and has no approved vaccine or antiviral drug [122–124]. Crowe et al. isolated CHKV-24, a super-neutralizing human-derived monoclonal antibody, from the B cells of a survivor naturally infected with CHIKV. The mRNAs encoding CHKV-24 antibodies were normally expressed in the sera of both, mice and nonhuman primates. This mRNA-LNP protected against CHIKV-induced arthritis, musculoskeletal diseases, and lethal attack in a mouse model and was well tolerated in crab-eating monkeys injected with CHKV-24 IgG. Notably, infusion of macaques with 0.5 mg/kg CHKV-24 mRNA achieved a mean maximal mAb concentration of 10.1–35.9 micrograms per milliliter, with a half-life of 23 days. This suggested that it may be feasible to treat humans by injecting CHIKV antibodies-encoding mRNAs [125]. Subsequently, Zaks et al. conducted a phase I human randomized placebo-controlled proof-of-concept trial between January 2019 and June 2020 to assess the safety and pharmacology of mRNA-1944 (mRNA-CHKV-24). The results revealed that two different mRNAs encoding the heavy and light chains of CHKV-24 IgG produced functionally neutralizing antibodies. In 28 actively treated participants, a single dose of mRNA-1944 at 0.1, 0.3, and 0.6 mg/kg resulted in a dose-related increase in CHKV-24 IgG serum levels. Peak CHKV-24 IgG levels exceeded 1 µg/mL at all doses with a overall mean terminal half-life (t1/2) of approximately 69 days. The mRNA-encoded antibodies could safely achieve the expected therapeutically relevant serum concentrations. This indicates that encoding different classes of antibodies by mRNAs has the same potential in treating various infectious diseases other than Chikungunya [88].

#### Defense against chronic hepatitis B virus by mRNA-encoded antibodies

The Hepatitis B virus (HBV) causes chronic hepatitis B (CHB). Globally, there are more than hundreds of millions of patients with chronic HBV infection [126, 127]. Ying et al. used mRNA drugs to encode three anti-HBsAg antibodies, namely, G12-scFv, G12-scFv-Fc, and G12-IgG genes, for the durable suppression of HBsAg. These mRNA drugs demonstrated better efficacy than exogenous G12 antibodies in an HBV-infected mouse model. Although the three mRNA drugs had different blood concentrations, terminal elimination half-life (t1/2), and EC50 of anti-HBs, they provided sustained and effective passive immunity in mice. A single dose of mRNA-G12-LNP in mice significantly reduced HBsAg serum levels within 30 days. In contrast, the exogenous antibodies lost their effect of reducing HBsAg levels after 9 days. These findings emphasize that antibody-encoding mRNAs have great potential to combat HBV infection [128, 129].

#### Defense against the respiratory syncytial virus by mRNA-encoded antibodies

Respiratory infections cause millions of hospitalizations and deaths worldwide each year. Palivizumab is a broad-spectrum neutralizing antibody against the respiratory syncytial virus (RSV) and the only FDA-approved treatment for high-risk populations [130–134]. Santangelo et al. were the first to express synthetic mRNAs encoding intact palivizumab (secreted form, called sPali) in the lungs via an endotracheal aerosol. Later, a glycosylated phosphatidylinositol (GPI) membrane anchor sequence from the decay-accelerating factor (DAF) was attached to the heavy chain mRNA of palivizumab (anchored form, termed aPali). Both sPali and aPali forms effectively prevented infection in a mouse RSV model, and most mRNA antibodies did not alter the baseline cytokine levels. Subsequently, the authors expressed two types of RSV VHH antibodies, namely, RSV aVHH and RSV sVHH, in a homogeneous manner. mRNA-aVHH significantly inhibited RSV 7 days after transfection, and it could be present in the lungs for at least 28 days, providing long-lasting protection to mice. RSV aVHH significantly inhibited RSV and persisted in the lungs for a long, providing long-lasting protection to mice. Overall, these data suggested that mRNA-encoded antibodies prevent RSV infection, with membrane-anchored antibodies exerting a more dramatic effect than the cellular forms [135].

### Tumor treatment by delivery of mRNAs encoding antibodies

#### Delivery of mRNAs encoding anti-PD-1 monoclonal antibody for tumor treatment

Immune checkpoints are a class of inhibitory molecules distributed on the surface of the cell membrane of immune cells, which transmit inhibitory signals to the immune cells by binding to ligands. When tumor cells express ligand molecules to interact with the immune checkpoints on the surface of the cell membrane, the immune system is not able to recognize these tumor cells, which contributes to the immune escape. The basic principle of immune checkpoint blockade therapy is to block the binding of immune checkpoints to their associated ligands through the use of immune checkpoint inhibitors (such as monoclonal antibody) [136–138]. PD-1 is an important immune checkpoint mainly expressed on activated T cells and binds to PD-L1 and PD-L2 ligands. Tumors evade immune cell pursuit by expressing PD-L1 on the cell surface and the PD-1/PD-L1 pathway is an important mechanism by which tumors promote immune escape [139, 140]. Pembrolizumab is an anti-PD-1 monoclonal antibody that exhibits significant antitumor efficacy and a favorable safety profile. It was approved for marketing by the FDA in 2014 [141, 142]. In June 2023, Moderna and Merck announce mRNA-4157 (V940) in combination with pembrolizumab demonstrated a statistically significant and clinically meaningful improvement in distant metastasis-free survival in patients with high-risk stage III/IV melanoma following complete resection versus pembrolizumab. This suggests that development of new oncology therapies based on pembrolizumab is possible [143, 144]. Shang et al. developed an LNP-based IVT-mRNA delivery system for delivering full-length pembrolizumab monoclonal antibodies. The results showed that in vitro pembrolizumab-mRNA has similar biological activities and functions in terms of affinity, binding specificity, blocking ability of PD-1 and PD-L1/L2, and enhancement of T cell function compared with commercial pembrolizumab. Following intravenous administration of a single dose of 2 mg/kg of mRNA-LNPs to mice, Pembrolizumab in serum exceeded 25 µg/mL for a duration of more than 35 days. In mouse experiments, the therapeutic effects of pembrolizumab-mRNAs were superior to that of protein-derived pembrolizumab. The tumors completely disappeared in five of the 10 mice injected with 2.0 mg/kg mRNA-LNPs, and there were no obvious side effects during the experiment. Moreover, in the assessment of therapeutic immunomodulation, flow cytometric analysis showed T cell activation in the experimental group. These results suggested that pembrolizumab-mRNA played a positive role in treating tumors [145]. Of course, although immune checkpoint blockade therapy is a promising tumor treatment method, it still has certain limitations. Immune checkpoint blockade therapy may cause immune-related adverse events (irAEs), hyperprogression or pseudoprogression [146]. Therefore, combination of multiple inhibitors may have better tumor-killing effects. For example, the marketed drug Opdualag (Nivolumab + Relatlimab) is combined with two immune checkpoint inhibitors, PD-1 and LAG-3, in order to obtain better anti-tumor effects [147].

#### Delivery of mRNAs encoding anti-HER2 monoclonal antibody for tumor treatment

Human epidermal growth factor receptor 2 (HER2) is a proto-oncogene with tyrosine kinase activity. Its overexpression leads to abnormal tumor cell proliferation [148, 149]. Trastuzumab is a humanized IgG1 monoclonal antibody that binds HER2 and exerts antitumor effects [150, 151]. Rybakova et al. designed and optimized an IVT mRNA delivery system using liver-targeted LNPs for in vivo delivery of trastuzumab-mRNA. According to the LC-MS analysis, the amino acid sequence of the trastuzumab-mRNA-encoded antibody was consistent with that of trastuzumab. Moreover, trastuzumab-mRNA had better pharmacokinetics than trastuzumab (Herceptin). Administration of 2 mg/kg mRNA to mice resulted in serum trastuzumab protein levels of approximately 40 mg/mL at 24 h, reaching 57.5 ± 7.6 mg/mL at 7 days post-injection. In contrast, serum clearance (Cl) and steady-state volume of distribution (Vss) were approximately 5-fold higher after injection with Herceptin than after injection of trastuzumab mRNA-LNPs at 30 days. Additionally, trastuzumab isolated from the sera of mice injected with IVT mRNA-LNP maintained specificity for HER2 as well as ADCC function. The full-sized antibodies produced in the livers of these mice effectively inhibited the growth of solid tumors as well as prolonged the survival of animals with HER2-positive breast cancer. Interestingly, the authors did not observe any significant toxic reactions during this process [152].

#### Delivery of mRNAs encoding bispecific antibodies to treat tumors

Bispecific antibodies are a bridge between target and functional cells, and stimulate a direct immune response; thus, they have promising applications in tumor immunotherapy. However, their complex manufacturing processes, short half-life, and harsh storage conditions greatly limit their clinical applications [110, 153, 154]. RiboMAbs are bispecific antibodies that bind CD3, a T-cell receptor-associated molecule, and three tumor-associated antigens (TAAs). Sahin et al. used a modified IVT mRNA for delivering RiboMAbs. This enabled sustained endogenous synthesis of antibodies that have higher terminal elimination half-lives than conventional antibodies. At the cellular level, RiboMAbs recruit peripheral blood mononuclear cells (PBMCs) to tumor cells and induce their activation ultimately causing tumor cell lysis. The RiboMAbs produced in vivo successfully eliminated the human breast cancer xenograft in NSG mice with better therapeutic efficacy than the same type of antibody purified in vitro. Notably, human PBMCs did not systemically release proinflammatory cytokines in the transplanted NSG mice, which suggests that non-specific T cell were not activated [91].

#### Delivery of mRNAs encoding bispecific antibodies in combination with other relevant drugs to treat tumors

Tumor-associated macrophages (TAMs) are highly concentrated and play an important role in immunosuppressing liver malignancies [155–157]. Liu et al. showed that CCL2 and CCL5 are important chemokines that trigger tumor-infiltrating monocytes in patients with hepatocellular carcinoma. Additionally, they attract TAM invasion and induce their differentiation to the pro-oncogenic M2 phenotype. The researchers designed a bispecific single-domain antibody for simultaneously blocking CCL2 and CCL5 (BisCCL2/5i) to reverse this immunosuppressive process. In their study, mRNAs encoding BisCCL2/5I were delivered to liver malignancies via liver-homing MC3 LNPs. A highly potent signal peptide at the N-terminal of BisCCL2/5I mediated efficient antibody secretion from the cytoplasm to the local tumor microenvironment (TME). These findings imply that BisCCL2/5i mRNA-LNPs can effectively polarize the pro-cancer M2 macrophages to the cancer-suppressive M1 macrophages and thus shift the TME to anti-tumor immunity. Interestingly, BisCCL2/5i mRNAs had a more significant effect than combined anti-CCL2 and anti-CCL5 antibodies or small molecule antagonists. Subsequently, the authors wrapped BisCCL2/5i and PD-1 ligand (PD-LI) antibody mRNAs in LNPs and administered it to mice in the same manner [158]. BisCCL2/5i along with PD-LI antibody produced a robust anti-cancer response in a mouse model of primary liver cancer as well as colorectal and pancreatic cancer with liver metastases, and achieved long-term survival of the samples. Further, the authors determined the applicability of BisCCL2/5i mRNA-LNP and PDLI mRNA-LNP delivery strategies by testing their safety in an in situ hepatocellular carcinoma model and observed no significant associated toxicities. These results suggested that the synergistic effect of dual blockade of CCL2/CCL5 by BisCCL2/5i mRNA-LNPs with PD-LI treatment can be extended to various secondary liver malignancies [159]. These findings provide a reference for the use of mRNA antibodies along with other drugs to treat the disease.

### Other medical applications for the delivery of mRNA encoding antibodies

In addition to the use of mRNA antibodies to prevent viral infections and treat tumors, some researchers have tried to apply mRNA antibodies in anti-toxin and anti-bacterial infections. Thran et al. presented the results of a BoNT/A challenge experiments, where mice injected intravenously with BoNT/A were quickly poisoned and died, whereas treatment with mRNA antibodies (VNA-BoNTA-LNP) within 6 h could effectively prevent death. This suggests that mRNA antibodies have potential antitoxin capabilities [160]. Moreover, a recent study by Moderna’s team showed that mRNA delivery of IgA type antibodies (mRNA-Sal4 and mRNA-CAM003) were effective in preventing infection of mice with *Salmonella* and *Pseudomonas aeruginosa*. IgA is challenging to translate clinically due to its highly glycosylated and protease-sensitive nature. Compared to recombinant protein antibodies, mRNA encoded IgA has a better pharmacokinetic profile, and this option may provide a novel idea for the development of short-lived protein drugs [161].

**Table 4:** Advances in the research of mRNA antibodies

References	[90]	[100]	[106]	[89]	[121]	[160]	[88]	[129]	[135]	[135]	[145]	[152]	[91]	[159]	[160]	[161]	[161]
Sponsor	Beijing Institute of Microbiology and Epidemiology	Wuhan Institute of Virology	University of Pennsylvania	VIB-UGent Center for Medical Biotechnology	Infectious Disease Research Institute	Tübingen	Moderna, Inc.	Fudan University	Georgia Institute of Technology and Emory University	Georgia Institute of Technology and Emory University	Dongtai People’s Hospital	Massachusetts Institute of Technology	BioNTech	niversity of North Carolina at Chapel Hill Chapel Hill	Tübingen	Moderna, Inc.	Moderna, Inc.
Dosege	0.2 and 1 mg/kg	5 × 10^5^ IU	1 and 1.4 mg/kg	5 µg	40 µg	40 µg	0.1, 0.3 and 0.6 mg/kg	2.5 mg/kg	20 and 100 µg	100 µg	0.2, 0.6 and 2 mg/kg	8 mg/kg	5 µg	1 mg/kg	40 µg	1 mg/kg	1 mg/kg
Route	I.V.	I.T.	I.V.	I.T.	I.M.	I.M.	I.V.	I.V.	I.T.	I.T.	I.V.	I.V.	I.V.	I.V.	I.V.	I.V.	I.V.
Main components of vector	DSPC, cholesterol and PEG-lipid		PEG-lipid, cholesterol, ionizable cationic lipid	DOTAP and cholesterol	Squalene, glyceryl trimyristate, DOTAP	Phosphatidylcholine, cholesterol, PEG-lipid	DSPC, cholesterol and PEG-lipid	DSPC, cholesterol, DMG-PEG2000			Phosphatidylcholine, PEGylated lipid cholesterol	cKK-E12	TransIT-mRNA Transfection kit	MC3 LNPs	Phosphatidylcholine, cholesterol, PEG-lipid	Undisclosed	Undisclosed
Indication	RBD	RBD	HIV-1 gp120	FcgRIV & M2e	ZIKV	Rabies	CHIKV	HBsAg	RSV	RSV	PD1	HER2	TAAs	CCL2 & CCL5	Botulinum toxin	O5-antigen	Psl
Diseases	SARS-CoV-2	SARS-CoV-2	HIV-1	Influenza A Virus	Zika Virus	Rabies	Chikungunya virus	Hepatitis B Virus	Respiratory syncytial virus	Respiratory syncytial virus	Oncology	Oncology	Oncology	Oncology	Toxins	STm	PA
Format	IgG	IgG	IgG	Bispecific	IgG	IgG	IgG	scFv, scFv-Fc, IgG	IgG	VHH	IgG	IgG	Bispecific	Bispecific	VNA	IgA2	IgA1
Antibodies	HB27	CB6	VRC01	RiboBiFE	ZIKV-117	S057(CR57)	mRNA-1944	G12	Palivizumab	RSV aVHH	Pembrolizumab	Trastuzumab	RiboMAbs	BisCCL2/5i	BoNT/A	Sal4	CAM003

## Discussion

The introduction of hybridoma technology in 1975 accelerated the development of the antibody industry and numerous researchers focused on this field. The monoclonal antibody technology has recently developed rapidly and is widely used in several biomedical and clinical fields [112, 162, 163]. After hybridoma technology, new methods of antibody preparation, such as phage display technology, natural whole human library technology, and single B-cell technology, have emerged. Although these methods continuously improve, antibody production costs are still high. Fortunately, the use of mRNA antibodies can overcome this challenge. The antibody-encoding mRNAs are transiently expressed by ribosomes and the antibodies produced can act directly on the patient; thus, greatly reducing the cost and duration of antibody production [11, 13].

Despite the advantages of mRNA antibodies, the mRNA delivery technology was not originally designed for passive immunotherapy. After almost > 30 years of development, mRNA biologics are being developed primarily for prophylactic vaccination against certain potentially infectious diseases or cancer [164]. The most typical case is COVID-19 prevention using the mRNA vaccines. Here, the spike protein-encoding mRNAs deliver antigenic information to the antigen-presenting cells for triggering the body’s immune response, which allows the body to develop resistance against pathogens [165–167]. The development of vaccines has played a significant role in preventing the spread of infectious diseases in humans. Nonetheless, this approach of delivering antigens for inducing active immunity in the body has certain limitations. For instance, antibody production to fight against invading pathogens takes time after the first vaccination, which can be fatal for some viruses at high risk of transmission [168]. Moreover, for patients who are already affected by a disease, vaccines that focus on prevention cannot solve the problem. In such situations, delivering highly effective monoclonal or bispecific antibodies via mRNA for rapidly inducing acquired immunity against viral or tumor cells is certainly a highly effective therapeutic approach [12, 169, 170]. However, the research on mRNA-delivered antibodies is currently limited and experimental data in large mammals are still lacking. Nevertheless, the entry of mRNA CHKV-24 (NCT03829384), a monoclonal antibody against chikungunya virus, into Phase I clinical trials has taken another substantial step towards treating human diseases [88].

Conclusively, mRNA antibody drugs are a therapeutic alternative to traditional protein antibodies; yet, they face many challenges to truly enter the public eye. In recent years, in addition to mRNA drugs, some drugs regarding non-coding RNAs (like microRNAs or siRNAs) have been under development. Certain non-coding RNAs are thought to be pervasive regulators of multiple cancer hallmarks. Moreover, non-coding RNAs can play a major role in resistance to different cancer therapies by reorganizing the necessary signaling pathways [171]. For example, an agent developed against miR-16 for the treatment of Malignant pleural mesothelioma and non-small cell lung cancer has now completed phase I clinical trials [172]. Delivery of non-coding RNAs can be achieved by nanovectors, viral transduction, introduction of chemical modifications, or binding to biomolecules to achieve effective intracellular delivery and thereby facilitate receptor-mediated uptake [173, 174]. Furthermore, how to optimize them for clinical applications is a major dilemma for researchers. With technological advancements and research progress, mRNA antibody drugs may truly enter people’s lives in the future.

## Data Availability

Not applicable.

## Abbreviations

IVT mRNA: In vitro transcribed mRNA
TLR: Toll-like receptor
HPLC: High Performance Liquid Chromatography
FPLC: Fast protein liquid chromatography
RNAP: RNA polymerase
VEEV: Venezuelan equine encephalitis virus
DOTAP: 1,2-Dioleoyl-3-trimethylammonium-propane
DSPC: 1, 2-distearoyl-sn-glycero-3-phosphocholine
S.C.: Subcutaneous injection
I.C.V.: Intracerebroventricular administration
I.V.: Intravenous injection
I.T.: Intratracheally administration
VHH: Variable domain of heavy chain of heavy chain antibody
scFv: Single chain antibody fragment
PD-1: Programmed cell death protein 1
PD-L1: Programmed cell death 1 ligand 1
LC-MS: Liquid Chromatograph Mass Spectrometer
ADCC: Antibody-dependent cell-mediated cytotoxicity
STm: *Salmonella enterica* serovar Typhimurium
PA: *Pseudomonas aeruginosa*
BoNT/A: Botulinum neurotoxin serotype A
VNA: VHH-based neutralizing agent
